# Finite-Bandwidth Protection of a Three-Level Quantum Heat Engine Against Parasitic Heat Leaks

**DOI:** 10.3390/e28101081

**Published:** 2026-09-30

**Authors:** Gilberto Aparecido Prataviera, Marcos César de Oliveira

**Affiliations:** 1 Departamento de Administração, Faculdade de Economia, Administração e Contabilidade de Ribeirão Preto (FEA-RP), Universidade de São Paulo, Ribeirão Preto 14040-905, SP, Brazil; prataviera@usp.br; 2 Instituto de Física Gleb Wataghin, Universidade Estadual de Campinas, Campinas 13083-859, SP, Brazil

**Keywords:** quantum heat engine, reservoir engineering, finite-bandwidth reservoir, parasitic heat leak, open quantum systems, GKSL master equation, Bloch–Redfield equation

## Abstract

Finite-bandwidth reservoir engineering can suppress unwanted transitions in a quantum thermal machine, but a physical filter also introduces a finite response time. We study this competition in a continuous three-level heat engine whose hot environment couples parasitically to the cold transition. The corresponding three-state rate network is solved exactly, showing that the parasitic transition produces a hot-to-cold thermodynamic short circuit and yielding a closed threshold for the loss of positive-power operation. We then retain a damped auxiliary mode explicitly as a physical spectral filter. Its Lorentzian response suppresses the detuned parasitic transition, whereas excessive narrowing limits the useful energy throughput. Independent local-GKSL and nonsecular Bloch–Redfield calculations both recover the no-leak Markovian engine in the weak-coupling limit and predict a finite maximum-power bandwidth, although its precise location is model dependent. A frequency-resolved Lorentzian-rate reduction, by contrast, has no interior optimum. The resulting design principle is that spectral selectivity can protect the useful thermodynamic cycle, but a real filter cannot be narrowed without a dynamical throughput cost.

## 1. Introduction

The three-level maser is a canonical model of a continuous quantum heat engine [1,2]. Its thermodynamic cycle can be expressed directly in terms of three transition frequencies. A hot reservoir excites the system through a transition of frequency $\omega_{h}$, a cold reservoir exchanges energy across a transition of frequency $\omega_{c}$, and the remaining transition,

(1) $$\epsilon =\omega_{h}-\omega_{c},$$

is connected to a work terminal. In the ideal tightly coupled model, every stationary cycle absorbs $\omega_{h}$ from the hot reservoir, releases $\omega_{c}$ to the cold reservoir, and transfers ϵ through the work terminal. The corresponding ideal efficiency is

(2) $$\eta_{0}=\frac{\epsilon }{\omega_{h}}.$$

Broader accounts of the theoretical and experimental developments in quantum heat engines and refrigerators can be found in Refs. [3,4,5] and references therein.

**Figure 1:**
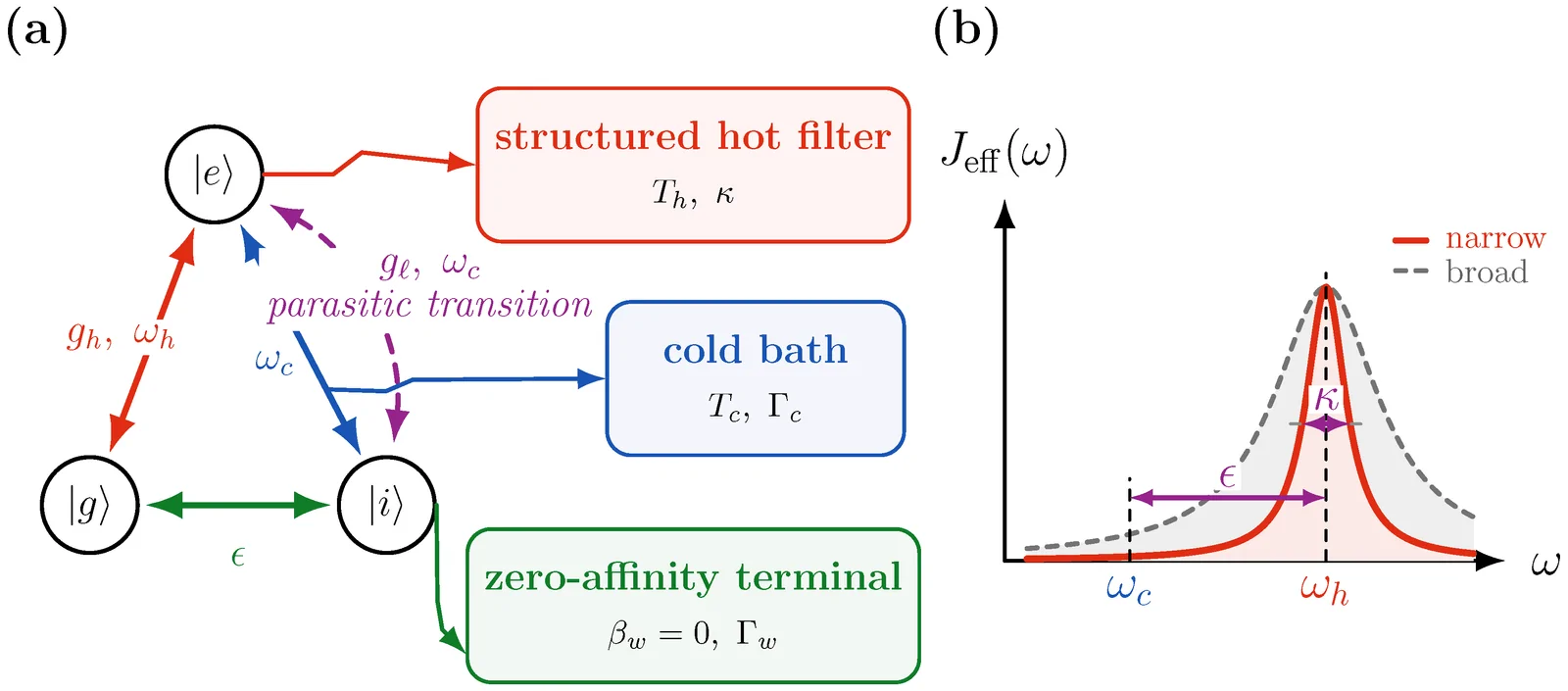
Engine cycle and finite-bandwidth protection. (**a**) The useful hot transition |g〉↔|e〉 is resonantly coupled to a structured hot filter, while the dashed purple line denotes the unwanted coupling of the same hot filter to the cold transition |i〉↔|e〉. The cold bath and the zero-affinity terminal act on the remaining two transitions. (**b**) Narrow and broad Lorentzian responses centered at $\omega_{h}$, shown at equal resonant height as imposed by $4g_{h}^{2}/\kappa =\Gamma_{h}$. Narrowing the filter suppresses the detuned response at $\omega_{c}=\omega_{h}-\epsilon$, while an excessively narrow filter becomes dynamically slow.

This picture assumes perfect transition selectivity. However, in an actual physical device, an environment designed to address one transition generally has a finite spectral width and can also drive a nearby transition. In the three-level engine considered here, the hot environment couples parasitically to the transition normally addressed by the cold bath (see Figure 1). The additional transition opens a second thermodynamic cycle that transfers heat from hot to cold without crossing the work transition, thereby breaking tight coupling and potentially reversing the useful cycle.

A natural strategy is to engineer a frequency-selective hot environment. Reaction-coordinate, pseudomode, and auxiliary resonator constructions provide physical realizations of structured reservoirs [6,7,8,9]. Related filtering ideas have been explored in quantum refrigerators and thermal management [10,11], and superconducting thermal circuits offer an experimentally relevant setting in which resonators shape the spectrum seen by a small quantum system [12,13].

The central issue is that spectral selectivity and dynamical response are not independent when the filter is a physical subsystem. A broad filter exchanges energy rapidly with its reservoir but poorly resolves the useful and parasitic transition frequencies. A narrow filter provides stronger spectral rejection, but its longer response time can itself limit the useful heat current. The competition between these two effects suggests that the best operating point may occur at a finite filter linewidth.

In this work, we establish how finite-bandwidth reservoir engineering can protect a continuous three-level quantum heat engine against a parasitic hot transition while accounting explicitly for the dynamical cost of the filter. We first formulate the three-level engine as an exactly solvable Markovian rate network including the parasitic hot transition. This allows us to prove that the parasitic transition generates a hot-to-cold thermodynamic short circuit and to derive a closed threshold for the loss of positive-power operation. We then replace the idealized frequency-dependent hot reservoir by an explicit damped auxiliary mode that acts as a physical spectral filter. Its finite linewidth suppresses the off-resonant parasitic transition, while its finite response time limits the useful energy throughput when the filter becomes too narrow.

The quantitative behavior of the resulting qutrit–filter system can depend on how its residual environments are modeled. We therefore compare several open-system descriptions. Our primary calculation uses a local Gorini–Kossakowski–Sudarshan–Lindblad (GKSL) master equation [14,15] for the enlarged system. As an independent robustness check, we also employ a frequency-resolved nonsecular Bloch–Redfield treatment in the dressed basis, retaining interference between near-degenerate transitions [16,17,18]. Both explicit filter descriptions are compared with a frequency-resolved Lorentzian-rate reduction obtained from the filter susceptibility. The latter can be connected analytically to the three-state model and, unlike the explicit dynamical descriptions, is shown not to possess an interior maximum of the stationary power. We additionally consider a fully secular global construction only as a diagnostic of the near-degenerate weak-coupling limit. The two explicit filter approaches independently predict a finite maximum-power bandwidth, although its precise location is model dependent. We further examine how this optimum changes with the parasitic coupling strength and thermal bias, and discuss a possible superconducting-circuit implementation using a strongly anharmonic artificial atom coupled to a tunable low-*Q* resonator.

The remainder of the paper is organized as follows. Section 2 solves the Markovian multicycle engine and derives the heat leak theorem and the engine breakdown threshold. Section 3 introduces the explicit finite-bandwidth filter and derives its time-domain response and Lorentzian spectrum. Section 4 presents the local-GKSL, nonsecular Redfield, and effective-rate descriptions on a common footing. Section 5 gives the numerical construction and the weak-coupling and Fock-space validation tests. Section 6 compares the bandwidth dependence and parameter trends predicted by the different methods. Section 7 discusses what is robust and what remains model dependent, and Section 8 summarizes the conclusions.

## 2. Three-Level Engine with a Parasitic Hot Transition

We begin by defining the three-level working medium and the transition rates that will be used throughout the analysis. We work in units in which $ћ=k_{B}=1$. The three energy eigenstates are denoted by |g〉, |i〉, and |e〉, with

(3) $$E_{g}=0,\;E_{i}=\epsilon ,\;E_{e}=\omega_{h},$$

so that the energy gap between |i〉 and |e〉 is

(4) $$\omega_{c}=\omega_{h}-\epsilon .$$

The useful hot transition is |g〉↔|e〉, the cold transition is |i〉↔|e〉, and the work transition is |g〉↔|i〉. In addition, the hot reservoir acts parasitically on the |i〉↔|e〉 transition. The geometry and spectral-filtering mechanism are summarized in Figure 1.

Let *a* and *b* denote, respectively, the hot excitation and relaxation rates on |g〉↔|e〉. Let $c_{c}$ and $d_{c}$ denote the cold excitation and relaxation rates on |i〉↔|e〉, and let $c_{\ell }$ and $d_{\ell }$ denote the additional excitation and relaxation rates produced by the hot reservoir on the same transition. The work terminal is represented by equal rates *w* in the two directions, corresponding to an unbiased (zero-affinity) channel. This channel is used operationally to define the stationary output power. It is not intended as a microscopic model of a battery or of ergotropic work storage.

The state population equations are given by

(5) $${\dot{p}}_{g}=-ap_{g}+bp_{e}+w\left(p_{i}-p_{g}\right),$$

(6) $${\dot{p}}_{i}=\left(d_{c}+d_{\ell }\right)p_{e}-\left(c_{c}+c_{\ell }\right)p_{i}+w\left(p_{g}-p_{i}\right),$$

(7) $${\dot{p}}_{e}=ap_{g}+\left(c_{c}+c_{\ell }\right)p_{i}-\left(b+d_{c}+d_{\ell }\right)p_{e}.$$

Probability conservation follows immediately by adding Equations (5)–(7).

It is useful to define the total upward and downward rates on the *i*–*e* transition,

(8) $$c=c_{c}+c_{\ell },\;d=d_{c}+d_{\ell }.$$

At stationarity, together with $p_{g}+p_{i}+p_{e}=1$, the linear equations give

(9) $$p_{g}=\frac{bc+w(b+d)}{Z},$$

(10) $$p_{i}=\frac{ad+w(b+d)}{Z},$$

(11) $$p_{e}=\frac{ac+w(a+c)}{Z},$$

where

(12) Z=ac+ad+bc+w(a+c+2b+2d).

We next identify the stationary current associated with the useful thermodynamic cycle.

The probability current through the work transition is

(13) $$I=w(p_{i}-p_{g}).$$

Substituting Equations (9) and (10) gives

(14) $$I=\frac{w\left[a\left(d_{c}+d_{\ell }\right)-b\left(c_{c}+c_{\ell }\right)\right]}{Z}.$$

At stationarity the same net useful-cycle current flows through the intended hot transition. The operational output power is therefore defined as

(15) P=ϵI.

Positive-power engine operation requires I>0. The direction of the current carried by the parasitic hot transition can be determined analytically from the same stationary solution.

The net probability current produced by the hot reservoir on the *i*–*e* transition is

(16) $$I_{\ell }=c_{\ell }p_{i}-d_{\ell }p_{e}.$$

For thermal reservoirs, detailed balance gives the ratios

(17) $$r_{h}=e^{-\beta_{h}\omega_{h}},\;r_{c}=e^{-\beta_{c}\omega_{c}},\;r_{\ell }=e^{-\beta_{h}\omega_{c}},$$

with

(18) $$a=br_{h},\;c_{c}=d_{c}r_{c},\;c_{\ell }=d_{\ell }r_{\ell }.$$

Using the stationary populations, Equation (16) becomes

(19) $$I_{\ell }=\frac{d_{\ell }}{Z}\left[bd_{c}r_{h}\left(r_{\ell }-r_{c}\right)+bw\left(r_{\ell }-r_{h}\right)+d_{c}w\left(r_{\ell }-r_{c}\right)\right].$$

The sign of this expression is fixed under ordinary engine conditions. Since $T_{h}>T_{c}$ at the same frequency $\omega_{c}$, one has $r_{\ell }>r_{c}$. Since $\omega_{h}>\omega_{c}$ at the same hot temperature, one also has $r_{\ell }>r_{h}$. All prefactors in Equation (19) are nonnegative, and therefore

(20) $$I_{\ell }>0.$$

With the sign convention of Equation (16), the parasitic hot current is always directed from the hot reservoir into the *i*–*e* transition. In the stationary network that injected energy is subsequently rejected through the cold channel. The unwanted transition is therefore a genuine hot-to-cold short circuit.

The corresponding energy currents are

(21) $$J_{h}=\omega_{h}I+\omega_{c}I_{\ell },$$

(22) $$J_{c}=-\omega_{c}\left(I+I_{\ell }\right),$$

and satisfy

(23) $$J_{h}+J_{c}-P=0,$$

which is the stationary first law of thermodynamics, as the energy absorbed from the hot reservoir is partitioned between the heat released to the cold reservoir and the extracted power. The efficiency in the positive-power regime is given by

(24) $$\eta =\frac{P}{J_{h}}=\frac{\eta_{0}}{1+\left(\omega_{c}/\omega_{h}\right)\left(I_{\ell }/I\right)}.$$

Equation (20) together with Equation (24) shows directly that a nonzero parasitic current lowers the efficiency below the tight-coupling value $\eta_{0}=\epsilon /\omega_{h}$.

The expression for the useful current also gives a closed condition for the loss of positive-power operation.

From Equation (14), the condition I>0 is

(25) $$a\left(d_{c}+d_{\ell }\right)>b\left(c_{c}+c_{\ell }\right).$$

Using Equation (18), this reduces to

(26) $$d_{c}\left(r_{h}-r_{c}\right)>d_{\ell }\left(r_{\ell }-r_{h}\right).$$

For bosonic reservoirs we write the downward rates as

(27) $$b=\frac{\Gamma_{h}}{1-r_{h}},\;d_{c}=\frac{\Gamma_{c}}{1-r_{c}},\;d_{\ell }=\frac{\Gamma_{\ell }}{1-r_{\ell }},$$

where $\Gamma_{h}$, $\Gamma_{c}$, and $\Gamma_{\ell }$ are the corresponding zero-temperature rate scales. The critical parasitic scale is then

(28) $$\Gamma_{\ell }^{\mathrm{crit}}=\Gamma_{c}\frac{\left(r_{h}-r_{c}\right)\left(1-r_{\ell }\right)}{\left(1-r_{c}\right)\left(r_{\ell }-r_{h}\right)}.$$

When $\Gamma_{\ell }>\Gamma_{\ell }^{\mathrm{crit}}$, the useful current changes sign.

In the absence of the parasitic channel, Equation (26) reduces to $r_{h}>r_{c}$, which is equivalent to

(29) $$\frac{T_{h}}{T_{c}}>\frac{\omega_{h}}{\omega_{c}}.$$

This is the usual positive-power condition for the ideal three-level engine. For the finite-bandwidth performance analysis presented in Section 6, we choose a representative parameter set well inside this engine regime and use $\omega_{h}$ as the energy scale, with

(30) $$\omega_{h}=1,\;\epsilon =0.4,\;\omega_{c}=0.6,$$

and

(31) $$\begin{array}{c} T_{h}=0.5,\;T_{c}=0.2,\\ \Gamma_{h}=\Gamma_{c}=0.01,\;\Gamma_{w}=0.001. \end{array}$$

These values satisfy $T_{h}/T_{c}=2.5>\omega_{h}/\omega_{c}=5/3$ and therefore correspond to positive-power operation in the absence of the parasitic transition. For this reference set, the critical parasitic rate scale is

(32) $$\Gamma_{\ell }^{\mathrm{crit}}=3.7932\times 10^{-3},$$

or approximately $0.38\;\Gamma_{h}$. Thus, a parasitic channel substantially weaker than the intended hot coupling can already alter the thermodynamic operation of the engine.

## 3. Explicit Finite-Bandwidth Filter

To implement frequency filtering dynamically, we replace the direct hot reservoir by an auxiliary harmonic mode centered at the useful transition frequency $\omega_{h}$. The enlarged Hamiltonian is

(33) $$\begin{array}{c} H=\omega_{h}\left|e\rangle \langle e\right|+\epsilon |i\rangle \langle i|+\omega_{h}a^{†}a\\ \;+g_{h}\left(|e\rangle \langle g|\;a+|g\rangle \langle e|\;a^{†}\right)\\ \;+g_{\ell }\left(|e\rangle \langle i|\;a+|i\rangle \langle e|\;a^{†}\right). \end{array}$$

Here, *a* is the annihilation operator of the auxiliary mode. The coupling $g_{h}$ is the microscopic qutrit–filter coupling associated with the useful |g〉↔|e〉 transition, which is resonant with the auxiliary mode. The coupling $g_{\ell }$ is the corresponding microscopic coupling of the same mode to the parasitic |i〉↔|e〉 transition. The latter transition is detuned from the filter resonance by

(34) $$\omega_{h}-\omega_{c}=\epsilon .$$

We parameterize the relative strength of the parasitic coupling by the dimensionless amplitude ratio

(35) $$\chi \equiv \frac{g_{\ell }}{g_{h}},$$

so that

(36) $$g_{\ell }=\chi g_{h}.$$

Thus, χ=1 corresponds to equal microscopic coupling amplitudes on the useful and parasitic transitions. This does not imply equal effective transition rates, because the two transitions occur at different frequencies relative to the filter resonance.

The auxiliary mode loses energy to a residual hot reservoir with linewidth κ. If $g_{h}$ were held fixed while κ were varied, changing the linewidth would also change the resonant weak-coupling rate. To isolate the effect of spectral width, we therefore impose the constant-Purcell prescription

(37) $$g_{h}=\frac{1}{2}\sqrt{\Gamma_{h}\kappa },$$

which keeps

(38) $$\frac{4g_{h}^{2}}{\kappa }=\Gamma_{h}$$

fixed throughout the bandwidth scan. For fixed χ, the parasitic microscopic coupling then scales proportionally,

(39) $$g_{\ell }=\frac{\chi }{2}\sqrt{\Gamma_{h}\kappa }.$$

Thus, $\Gamma_{h}$ sets the intended resonant weak-coupling scale, while χ fixes the ratio of the two microscopic qutrit–filter coupling amplitudes.

The frequency selectivity and finite response time of the auxiliary mode follow directly from its Langevin equation. For clarity, consider first the useful transition operator $A_{h}\equiv \left|g\rangle \langle e\right|$. The mode obeys

(40) $$\dot{a}\left(t\right)=-\left(\frac{\kappa }{2}+i\omega_{h}\right)a\left(t\right)-ig_{h}A_{h}\left(t\right)+\sqrt{\kappa }\;a_{\mathrm{in}}\left(t\right),$$

where $a_{\mathrm{in}}\left(t\right)$ is the input noise of the residual hot bath. The formal solution is

(41) $$\begin{array}{c} a(t)=e^{-(\kappa /2+i\omega_{h})t}a\left(0\right)\\ \;-ig_{h}\int_{0}^{t}ds\;e^{-\left(\kappa /2+i\omega_{h}\right)\left(t-s\right)}A_{h}\left(s\right)\\ \;+\sqrt{\kappa }\int_{0}^{t}ds\;e^{-\left(\kappa /2+i\omega_{h}\right)\left(t-s\right)}a_{\mathrm{in}}\left(s\right). \end{array}$$

For a Markovian thermal input at temperature $T_{h}$, the nonvanishing noise correlations are

(42) $$\langle a_{\mathrm{in}}^{†}\left(t\right)a_{\mathrm{in}}\left(t^{\prime }\right)\rangle =n_{h}\delta \left(t-t^{\prime }\right),$$

(43) $$\langle a_{\mathrm{in}}\left(t\right)a_{\mathrm{in}}^{†}\left(t^{\prime }\right)\rangle =\left(n_{h}+1\right)\delta \left(t-t^{\prime }\right),$$

where

(44) $$n_{h}=\frac{1}{e^{\omega_{h}/T_{h}}-1},$$

and $\langle a_{\mathrm{in}}\left(t\right)a_{\mathrm{in}}\left(t^{\prime }\right)\rangle =\langle a_{\mathrm{in}}^{†}\left(t\right)a_{\mathrm{in}}^{†}\left(t^{\prime }\right)\rangle =0$. These correlations are the Langevin counterpart of the thermal auxiliary-mode dissipator introduced in Equation (53).

The retarded response is therefore proportional to

(45) $$\chi_{a}\left(\tau \right)=\Theta \left(\tau \right)e^{-\kappa \tau /2}e^{-i\omega_{h}\tau },$$

so the characteristic filter response time is of order 2/κ, and Θ(τ) is the step function. Its Fourier transform is given by

(46) $${\tilde{\chi }}_{a}\left(\omega \right)=\frac{1}{\kappa /2-i(\omega -\omega_{h})},$$

and the corresponding dissipative response is

(47) $$J_{\mathrm{eff}}\left(\omega \right)=g_{h}^{2}\kappa {\left|{\tilde{\chi }}_{a}\left(\omega \right)\right|}^{2}=\frac{g_{h}^{2}\kappa }{{\left(\kappa /2\right)}^{2}+{\left(\omega -\omega_{h}\right)}^{2}}.$$

At resonance, Equations (38) and (47) give

(48) $$J_{\mathrm{eff}}\left(\omega_{h}\right)=\Gamma_{h}.$$

At the parasitic frequency $\omega_{c}=\omega_{h}-\epsilon$,

(49) $$\frac{J_{\mathrm{eff}}\left(\omega_{c}\right)}{J_{\mathrm{eff}}\left(\omega_{h}\right)}=\frac{\kappa^{2}}{\kappa^{2}+4\epsilon^{2}}.$$

Equation (49) is useful for interpreting the numerical results. It quantifies the purely spectral benefit of reducing the linewidth.

For the local-GKSL maximum-power point reported in Equation (89), $\kappa_{★}=0.136443$, Equation (49) gives

(50) $$\frac{J_{\mathrm{eff}}\left(\omega_{c}\right)}{J_{\mathrm{eff}}\left(\omega_{h}\right)}=2.83\times 10^{-2}.$$

Thus, the filter suppresses the detuned spectral weight to about 2.8% of its resonant value at the maximum-power point.

The same parameter κ controls both spectral width and dynamical response. In the narrow-bandwidth limit, Equation (37) gives $g_{h}\propto \sqrt{\kappa }$, while the residual hot bath dissipator introduced in Section 4.1 is proportional to κ. The auxiliary mode therefore becomes weakly replenished by the hot reservoir and weakly coupled to the qutrit. The stationary hot current and output power consequently vanish as the filter is made sufficiently narrow.

At larger κ, the filter exchanges energy more rapidly with the hot reservoir. However, the suppression factor in Equation (49) increases, so the parasitic transition is progressively reactivated. The power is therefore expected to increase from zero at small bandwidth, reach a maximum at an intermediate linewidth, and eventually decrease as spectral selectivity is lost. The explicit filter results presented in Section 6 test this expectation without eliminating the auxiliary mode.

## 4. Open-System Descriptions and Effective Reduction

We now consider three complementary open-system descriptions. The local-GKSL and nonsecular Redfield approaches retain the enlarged qutrit–filter Hamiltonian *H*, while the effective Lorentzian-rate model is formulated for the reduced three-level system with Hamiltonian $H_{A}$. All three descriptions use the same reservoir temperatures, coupling ratio χ, and constant-Purcell protocol; they differ in how the filter dynamics and residual environments are incorporated into the stationary description.

### 4.1. Local GKSL Equation: Primary Model

Our primary model treats the residual hot bath, cold bath, and zero-affinity terminal as weakly coupled Markovian environments acting on the physical filter and bare qutrit transitions. The enlarged density operator obeys

(51) $$\dot{\rho }=-i\left[H,\rho \right]+L_{h}\rho +L_{c}\rho +L_{w}\rho ,$$

with

(52) $$D\left[L\right]\rho =L\rho L^{†}-\frac{1}{2}\left\{L^{†}L,\rho \right\}.$$

The residual hot bath acts on the auxiliary mode,

(53) $$L_{h}\rho =\kappa \left(n_{h}+1\right)D\left[a\right]\rho +\kappa n_{h}D\left[a^{†}\right]\rho ,$$

where $n_{h}$ is given in Equation (44). The cold bath acts on the bare *e*–*i* transition,

(54) $$\begin{array}{c} L_{c}\rho =\Gamma_{c}\left(n_{c}+1\right)D[|i\rangle \langle e|]\rho +\Gamma_{c}n_{c}D[|e\rangle \langle i|]\rho , \end{array}$$

with $n_{c}={\left(e^{\omega_{c}/T_{c}}-1\right)}^{-1}$, and the zero-affinity terminal is

(55) $$L_{w}\rho =\Gamma_{w}D[|g\rangle \langle i|]\rho +\Gamma_{w}D[|i\rangle \langle g|]\rho .$$

This local construction has a direct physical interpretation and is of GKSL form, so positivity is preserved by construction. Its quantitative scope is that of a weak-coupling local master equation: the residual-bath rates must remain small compared with the bare transition frequencies, while the coherent couplings $g_{h}$ and $g_{\ell }$ are retained exactly inside *H*.

### 4.2. Nonsecular Bloch–Redfield Equation: Independent Dressed-Basis Check

To test whether the finite-bandwidth optimum depends qualitatively on the local dissipative construction, we independently treat the same enlarged Hamiltonian with a frequency-resolved nonsecular Bloch–Redfield equation. Let

(56) $$H|n\rangle =E_{n}|n\rangle$$

and define the Hermitian coupling operators

(57) $$S_{h}=a+a^{†},\;S_{c}=\left|e\rangle \langle i\right|+|i\rangle \langle e|,\;S_{w}=\left|i\rangle \langle g\right|+|g\rangle \langle i|.$$

For terminal ν we introduce

(58) $$G_{\nu }=\sum_{m,n}\gamma_{\nu }\left(E_{n}-E_{m}\right)\langle m|S_{\nu }|n\rangle |m\rangle \langle n|,$$

and use

(59) $$R_{\nu }\left[\rho \right]=\frac{1}{2}(G_{\nu }\rho S_{\nu }+S_{\nu }\rho G_{\nu }^{†}-S_{\nu }G_{\nu }\rho -\rho G_{\nu }^{†}S_{\nu }).$$

For the residual hot and cold baths the rates obey thermal detailed balance,

(60) $$\gamma_{\nu }\left(\omega \right)=\left\{\begin{array}{cc} \Gamma_{\nu }\left[n_{\nu }\left(\omega \right)+1\right], & \omega >0,\\ \Gamma_{\nu }n_{\nu }\left(|\omega |\right), & \omega <0, \end{array}\right.$$

with $\Gamma_{h}$ replaced by κ for the residual hot bath. The work terminal is assigned a flat zero-affinity rate $\gamma_{w}\left(\omega \right)=\Gamma_{w}$ on the energy-exchanging dressed transitions. Equation (59) retains cross terms between different dressed Bohr frequencies and is therefore not fully secularized.

The Redfield generator is not GKSL in general. We therefore use it only as an independent stationary robustness check and explicitly verify positivity, first-law energy balance, and second-law Clausius consistency at the representative optima reported in Section 6.3. The advantage is that the residual baths are evaluated at the actual dressed frequencies while interference between nearly degenerate transitions is not removed by hand.

### 4.3. Effective Lorentzian-Rate Model and Fully Secular Control

The relation between the effective-rate description and the exact three-state model of Section 2 can be made explicit through a frequency-resolved weak-coupling reduction of the structured hot environment. The Lorentzian rate scale follows from eliminating the auxiliary mode at the level of its susceptibility. Using the filter susceptibility derived in Section 3, Equation (46), the resonant |g〉↔|e〉 transition acquires the effective hot rate scale

(61) $$\Gamma_{h}^{\mathrm{eff}}=\frac{4g_{h}^{2}}{\kappa }=\Gamma_{h},$$

where the last equality follows from the constant-Purcell prescription of Equation (38).

For the parasitic |i〉↔|e〉 transition, whose detuning from the filter resonance is $\omega_{c}-\omega_{h}=-\epsilon$, the corresponding effective rate is

(62) $$\Gamma_{\ell }\left(\kappa \right)=\frac{g_{\ell }^{2}\kappa }{{\left(\kappa /2\right)}^{2}+\epsilon^{2}}=\frac{\chi^{2}g_{h}^{2}\kappa }{{\left(\kappa /2\right)}^{2}+\epsilon^{2}}=\chi^{2}\Gamma_{h}\frac{\kappa^{2}}{\kappa^{2}+4\epsilon^{2}}.$$

Thus, the Lorentzian suppression obtained in Section 3 translates, after adiabatic elimination, into a bandwidth-dependent parasitic transition rate.

The same complex filter response also generates a dispersive correction. With the present detuning convention,

(63) $$\Delta_{\ell }\left(\kappa \right)=-\frac{g_{\ell }^{2}\epsilon }{{\left(\kappa /2\right)}^{2}+\epsilon^{2}}=-\frac{\chi^{2}g_{h}^{2}\epsilon }{{\left(\kappa /2\right)}^{2}+\epsilon^{2}}=-\chi^{2}\Gamma_{h}\frac{\kappa \epsilon }{\kappa^{2}+4\epsilon^{2}}.$$

This term may be interpreted as a filter-induced shift of the atomic transition. As in the other stationary calculations of the present work, we neglect this Hamiltonian renormalization and retain the dissipative contribution in Equation (62).

To connect the eliminated filter directly with the thermal rate model of Section 2, we now use a frequency-resolved adiabatic description in which the thermal occupation of the hot reservoir is evaluated at the frequency of each atomic transition. The resulting reduced master equation for the three-level system is

(64) $$\begin{array}{c} {\dot{\rho }}_{A}=-i[H_{A},\rho_{A}]\\ \;+\Gamma_{h}\left[n_{h}\left(\omega_{h}\right)+1\right]D\left[\sigma_{ge}\right]\rho_{A}+\Gamma_{h}n_{h}\left(\omega_{h}\right)D\left[\sigma_{eg}\right]\rho_{A}\\ \;+\Gamma_{\ell }\left(\kappa \right)\left[n_{h}\left(\omega_{c}\right)+1\right]D\left[\sigma_{ie}\right]\rho_{A}+\Gamma_{\ell }\left(\kappa \right)n_{h}\left(\omega_{c}\right)D\left[\sigma_{ei}\right]\rho_{A}\\ \;+\Gamma_{c}\left[n_{c}\left(\omega_{c}\right)+1\right]D\left[\sigma_{ie}\right]\rho_{A}+\Gamma_{c}n_{c}\left(\omega_{c}\right)D\left[\sigma_{ei}\right]\rho_{A}\\ \;+\Gamma_{w}D\left[\sigma_{gi}\right]\rho_{A}+\Gamma_{w}D\left[\sigma_{ig}\right]\rho_{A}, \end{array}$$

where

(65) $$H_{A}=\omega_{h}\left|e\rangle \langle e\right|+\epsilon |i\rangle \langle i|,$$

$\sigma_{\alpha \beta }=\left|\alpha \rangle \langle \beta \right|$, and

(66) $$n_{\nu }\left(\omega \right)=\frac{1}{e^{\omega /T_{\nu }}-1}.$$

Equation (64) should be understood as the frequency-resolved adiabatic reduction of the structured hot environment. It is distinct from a direct elimination of the local dissipator in Equation (53), for which the auxiliary-mode thermal occupation is fixed at $\omega_{h}$. The frequency-resolved form is the appropriate reduced description for establishing the connection with the thermal detailed-balance structure of Section 2.

The rates appearing in the population equations can then be identified directly with those introduced in Section 2:

(67) $$a=\Gamma_{h}n_{h}\left(\omega_{h}\right),\;b=\Gamma_{h}\left[n_{h}\left(\omega_{h}\right)+1\right],$$

(68) $$c_{c}=\Gamma_{c}n_{c}\left(\omega_{c}\right),\;d_{c}=\Gamma_{c}\left[n_{c}\left(\omega_{c}\right)+1\right],$$

(69) $$c_{\ell }\left(\kappa \right)=\Gamma_{\ell }\left(\kappa \right)n_{h}\left(\omega_{c}\right),\;d_{\ell }\left(\kappa \right)=\Gamma_{\ell }\left(\kappa \right)\left[n_{h}\left(\omega_{c}\right)+1\right],$$

(70) $$w=\Gamma_{w}.$$

They satisfy

(71) $$\frac{a}{b}=e^{-\beta_{h}\omega_{h}},\;\frac{c_{c}}{d_{c}}=e^{-\beta_{c}\omega_{c}},\;\frac{c_{\ell }}{d_{\ell }}=e^{-\beta_{h}\omega_{c}},$$

which are precisely the detailed-balance relations used in Equation (18). Consequently, the population sector of the frequency-resolved adiabatic master equation reduces exactly to Equations (5)–(7), with the bandwidth dependence entering through $\Gamma_{\ell }\left(\kappa \right)$. The stationary populations, currents, efficiency, and engine-reversal condition obtained analytically in Section 2 can therefore be used directly for the effective Lorentzian-rate model.

This correspondence gives two additional analytical consequences. First, let

(72) $$x\left(\kappa \right)\equiv d_{\ell }\left(\kappa \right)=\frac{\Gamma_{\ell }\left(\kappa \right)}{1-r_{\ell }},$$

and define

(73) $$A=d_{c}\left(r_{h}-r_{c}\right),\;B=r_{\ell }-r_{h}.$$

In the engine regime considered here, A>0 and B>0. Equation (14) can then be written as

(74) $$I\left(\kappa \right)=\frac{wb\;[A-Bx(\kappa )]}{Z_{0}+Z_{1}x\left(\kappa \right)},$$

where

(75) $$Z_{0}=ad_{c}r_{c}+ad_{c}+bd_{c}r_{c}+w\left(a+d_{c}r_{c}+2b+2d_{c}\right),$$

(76) $$Z_{1}=a\left(1+r_{\ell }\right)+br_{\ell }+w\left(2+r_{\ell }\right).$$

Since $Z_{0}>0$ and $Z_{1}>0$,

(77) $$\frac{\partial I}{\partial x}=-\frac{wb\left(BZ_{0}+AZ_{1}\right)}{{\left(Z_{0}+Z_{1}x\right)}^{2}}<0.$$

Moreover,

(78) $$\frac{d\Gamma_{\ell }}{d\kappa }=\frac{8\chi^{2}\Gamma_{h}\epsilon^{2}\kappa }{{\left(\kappa^{2}+4\epsilon^{2}\right)}^{2}}>0.$$

It follows that, within the adiabatically eliminated rate model,

(79) $$\frac{dP_{\mathrm{rate}}}{d\kappa }<0$$

throughout the positive-power regime. Hence, the reduced rate model cannot possess an interior maximum of the stationary power as a function of κ. Formally, narrowing the filter always improves the rate-model performance by suppressing the parasitic channel. The turnover obtained when the auxiliary mode is retained explicitly therefore originates from the dynamical response of the physical filter, which is absent after adiabatic elimination.

Second, the engine-reversal threshold derived in Section 2 can be converted into an analytical critical bandwidth. Setting

(80) $$\Gamma_{\ell }\left(\kappa_{\mathrm{rev}}\right)=\Gamma_{\ell }^{\mathrm{crit}}$$

and using Equation (62) gives

(81) $$\kappa_{\mathrm{rev}}=2\epsilon \sqrt{\frac{\Gamma_{\ell }^{\mathrm{crit}}}{\chi^{2}\Gamma_{h}-\Gamma_{\ell }^{\mathrm{crit}}}},$$

provided $\chi^{2}\Gamma_{h}>\Gamma_{\ell }^{\mathrm{crit}}$. If $\chi^{2}\Gamma_{h}\leq \Gamma_{\ell }^{\mathrm{crit}}$, the effective parasitic rate remains below the reversal threshold for all bandwidths.

The adiabatic description should, however, be interpreted within its regime of validity. Under the constant-Purcell prescription,

(82) $$\frac{g_{h}}{\kappa }=\frac{1}{2}\sqrt{\frac{\Gamma_{h}}{\kappa }},$$

which increases as the filter is narrowed. Consequently, sufficiently small κ eventually lies outside the regime in which the auxiliary mode can be regarded as instantaneously following the qutrit. The formal boundary optimum κ→0 of the rate model should therefore not be interpreted as a controlled prediction of the adiabatic approximation in that limit. It instead emphasizes precisely why the explicit dynamical treatment of the auxiliary mode is required to describe the narrow-bandwidth regime.

For completeness, we also tested a fully secular global generator built from the dressed transitions of *H*, whose construction is given explicitly in Appendix C. It is completely positive, but in the present near-degenerate weak-coupling limit it does not recover the elementary no-leak three-state engine. We therefore show it only in the weak-coupling validation test and do not use it for quantitative performance predictions.

### 4.4. Currents, Power, and Stationary Consistency

For the local GKSL description the stationary state satisfies

(83) $$0=-i\left[H,\rho_{\mathrm{ss}}\right]+\sum_{\nu }L_{\nu }\rho_{\mathrm{ss}},$$

and the terminal currents are

(84) $$J_{\nu }=\mathrm{Tr}\;\left[H\;L_{\nu }\left(\rho_{\mathrm{ss}}\right)\right].$$

For Redfield, we replace $L_{\nu }$ by $R_{\nu }$ in the same definition. With $J_{\nu }>0$ denoting energy supplied to the enlarged system,

(85) $$P=-J_{w},\;\eta =\frac{P}{J_{h}},\;J_{h}>0,\;P>0.$$

At stationarity, the terminal currents must satisfy the first-law energy balance

(86) $$J_{h}+J_{c}+J_{w}=0,$$

which is equivalent to $J_{h}+J_{c}-P=0$ because $P=-J_{w}$. To assess consistency with the second law of thermodynamics, we also monitor the stationary Clausius entropy-production quantity

(87) $${\dot{\Sigma }}_{C}=-\frac{J_{h}}{T_{h}}-\frac{J_{c}}{T_{c}}.$$

For thermodynamically consistent operation, one expects ${\dot{\Sigma }}_{C}\geq 0$. For the local GKSL model, however, this quantity is used here as a stationary second-law consistency diagnostic rather than as a general theorem for local master equations; for Redfield, it is likewise a diagnostic rather than a consequence of complete positivity.

## 5. Numerical Construction and Validation

The oscillator is truncated to the first *N* Fock states, giving Hilbert-space dimension 3N. For each κ and χ, we set $g_{h}=\sqrt{\Gamma_{h}\kappa }/2$, $g_{\ell }=\chi g_{h}$, and construct *H*, build the chosen dissipative generator, vectorize the stationary equation, replace one row by $\mathrm{Tr}\rho_{\mathrm{ss}}=1$, and solve the resulting linear system. The local-GKSL vectorization is given in Appendix A; the Redfield construction is summarized in Appendix B.

**Figure 2:**
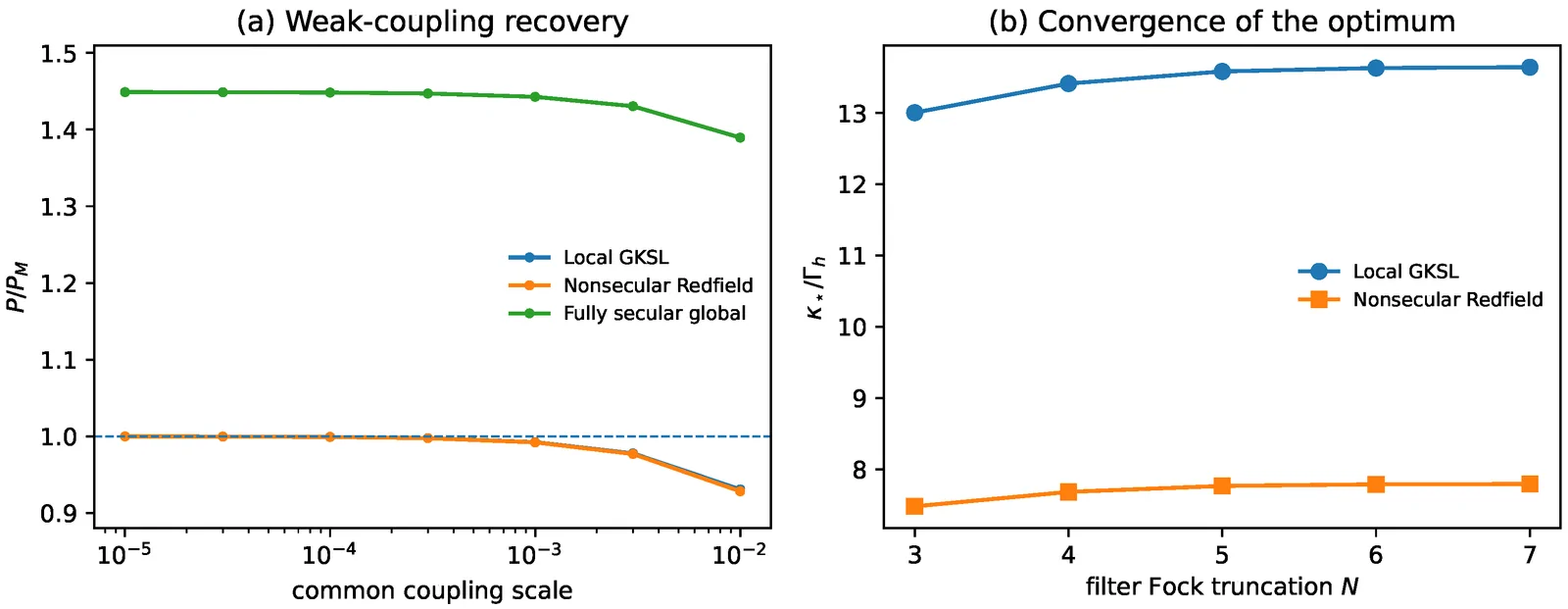
Validation of the dynamical descriptions. (**a**) Recovery of the exact no-leak Markovian engine as the common coupling scale Γ is reduced at χ=0 and κ=0.1. The local GKSL and nonsecular Redfield treatments approach $P/P_{M}=1$ marked by the blue dashed line, whereas the fully secular global construction does not. (**b**) Convergence of the maximum-power bandwidth with filter Fock truncation for χ=1. Both retained explicit filter descriptions converge rapidly, although to different quantitative optima.

A necessary check is recovery of the elementary no-leak Markovian engine. We set χ=0, κ=0.1 and scale

(88) $$\Gamma_{h}=\Gamma_{c}=\Gamma ,\;\Gamma_{w}=\Gamma /10$$

toward zero. Figure 2a shows that both the local GKSL and nonsecular Redfield results approach the exact Markovian power $P_{M}$ and reach $P/P_{M}=0.999850$ and 0.999847, respectively, at $\Gamma =10^{-5}$. By contrast, the fully secular global control approaches approximately 1.449 rather than unity. This failure is the reason it is not retained as a quantitative model here.

Figure 2b shows the independent Fock-space convergence of the two retained explicit filter descriptions. From N=6 to N=7, the local-GKSL optimum changes from 13.6332 to 13.6443 in units of $\Gamma_{h}$, while the Redfield optimum changes from 7.7887 to 7.7938. At the local-GKSL optimum the mean filter occupation is 0.15541 and the highest retained state n=6 carries only $5.14\times 10^{-6}$ probability.

## 6. Finite-Bandwidth Protection: Comparison of Methods

Unless otherwise stated we use $\omega_{h}=1$, ϵ=0.4, $\omega_{c}=0.6$, $T_{h}=0.5$, $T_{c}=0.2$, $\Gamma_{h}=\Gamma_{c}=0.01$, $\Gamma_{w}=0.001$, and N=7.

### 6.1. Bandwidth Dependence

**Figure 3:**
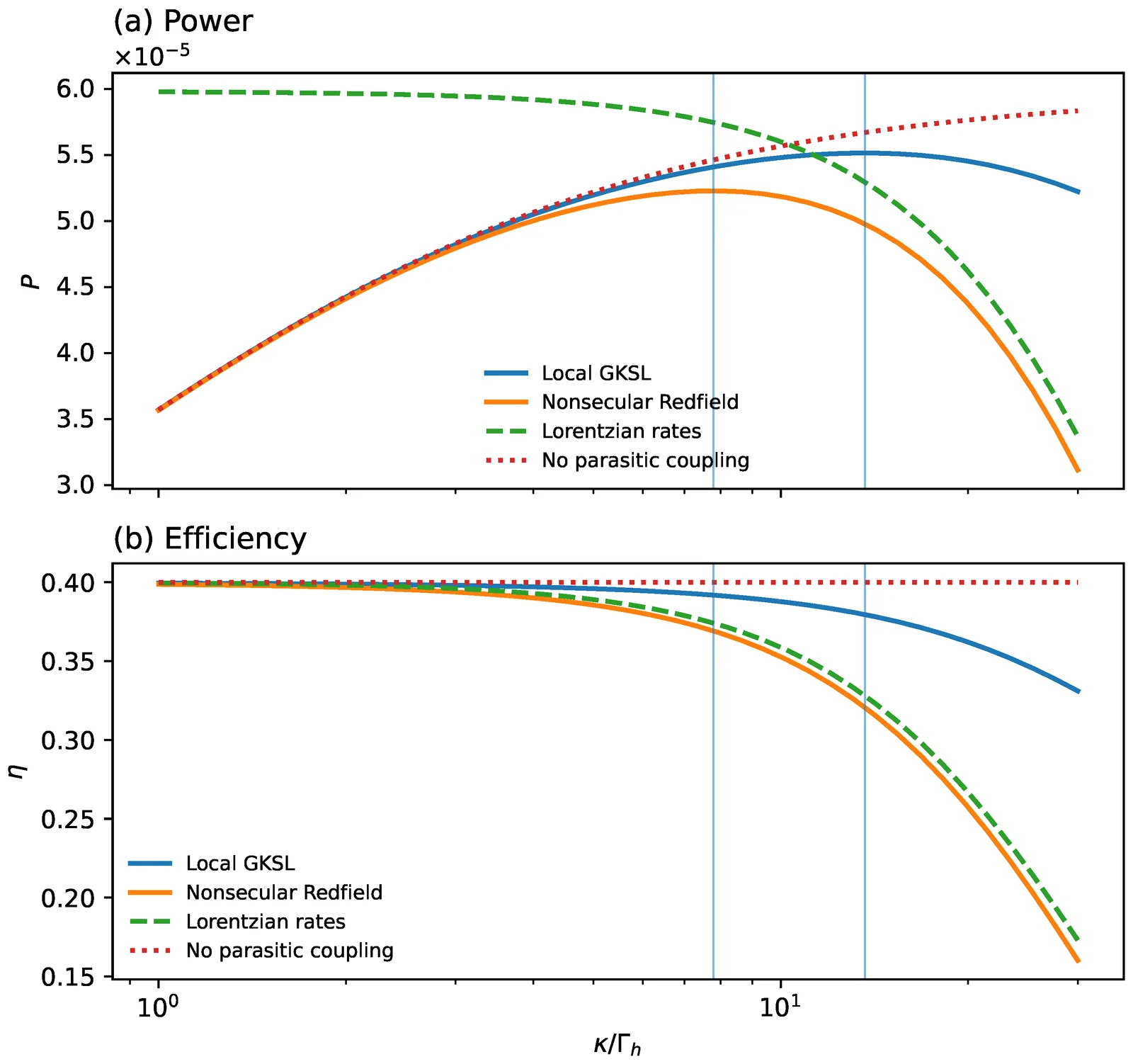
Method comparison for χ=1. (**a**) Stationary power and (**b**) efficiency versus filter bandwidth. The local GKSL equation is the primary explicit filter model; the nonsecular Redfield curve is an independent frequency-resolved check; the Lorentzian-rate curve is the frequency-resolved effective-rate reduction; and the no-parasitic curve is the local-GKSL reference at the same bandwidth. The two thin vertical markers indicate the Redfield and GKSL maximum-power bandwidths, respectively. The displayed range is restricted to κ≤0.3.

Figure 3 compares all retained descriptions for χ=1. The local-GKSL and nonsecular Redfield calculations both exhibit an interior maximum of the stationary power. By contrast, the Lorentzian-rate result decreases monotonically with κ, as proved analytically in Equation (79), and therefore has no interior maximum.

The converged local-GKSL maximum is

(89) $$\begin{array}{c} \frac{\kappa_{★}^{\mathrm{GKSL}}}{\Gamma_{h}}=13.6443,\\ P_{★}^{\mathrm{GKSL}}=5.5153\times 10^{-5},\;\eta_{★}^{\mathrm{GKSL}}=0.37950. \end{array}$$

At the same bandwidth the local-GKSL engine with the parasitic coupling removed has $P_{0}=5.6709\times 10^{-5}$ and $\eta_{0}=0.4000$, hence

(90) $$\frac{P_{★}^{\mathrm{GKSL}}}{P_{0}}=0.9726,\;\frac{\eta_{★}^{\mathrm{GKSL}}}{\eta_{0}}=0.9488.$$

The corresponding spectral suppression is $J_{\mathrm{eff}}\left(\omega_{c}\right)/J_{\mathrm{eff}}\left(\omega_{h}\right)=2.83\times 10^{-2}$.

The independent Redfield calculation gives

(91) $$\begin{array}{c} \frac{\kappa_{★}^{R}}{\Gamma_{h}}=7.7938,\\ P_{★}^{R}=5.2288\times 10^{-5},\;\eta_{★}^{R}=0.36913. \end{array}$$

At its own optimum the corresponding no-leak Redfield calculation gives $P_{0}^{R}=5.4528\times 10^{-5}$ and $\eta_{0}^{R}=0.39982$, so the retained fractions are 0.9589 in power and 0.9232 in efficiency. The numerical optimum therefore shifts appreciably between dissipative constructions, but the finite optimum, the protected high-performance regime, and the underlying bandwidth competition survive.

### 6.2. Robustness with Parasitic Coupling and Thermal Bias

**Figure 4:**
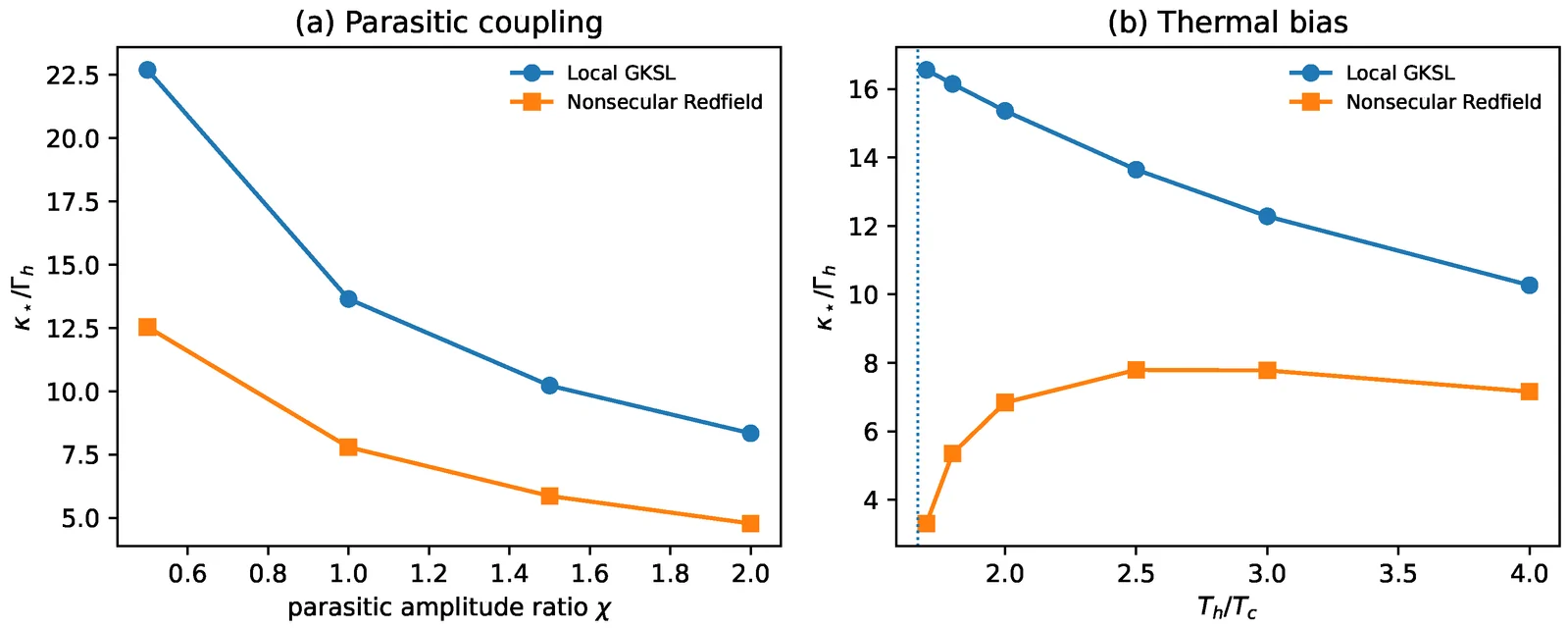
Robustness of the optimal-bandwidth trend. (**a**) Maximum-power bandwidth versus parasitic microscopic amplitude ratio χ. (**b**) Maximum-power bandwidth versus thermal bias at fixed $T_{c}=0.2$ and χ=1. The local GKSL and nonsecular Redfield descriptions differ quantitatively but predict the same finite-bandwidth protection mechanism and the same shift toward narrower filters as the parasitic coupling is increased. The vertical dotted line in panel (**b**) marks the no-leak threshold $T_{h}/T_{c}=5/3$.

A useful robustness criterion is whether the two explicit filter descriptions predict the same systematic movement of the optimum as physical parameters are changed. Figure 4a shows that they do. As χ increases from 0.5 to 2, the GKSL optimum moves from $\kappa_{★}/\Gamma_{h}=22.69$ to 8.34, while the Redfield optimum moves from 12.54 to 4.78. Stronger parasitic coupling therefore consistently favors a narrower filter.

The thermal-bias comparison in Figure 4b is likewise informative. In the primary GKSL model the optimum decreases monotonically from 16.56 at $T_{h}/T_{c}=1.7$ to 10.27 at $T_{h}/T_{c}=4$. Redfield predicts a stronger near-threshold shift but again a finite optimum throughout the engine regime, with $\kappa_{★}/\Gamma_{h}=7.79$ at the representative ratio 2.5. The exact numerical ridge is therefore method dependent, especially close to threshold, whereas the existence of a finite protected operating scale is not. Some comparative values are given in Table 1.

### 6.3. Stationary Thermodynamic Checks

At the local-GKSL optimum the currents are

(92) $$\begin{array}{c} J_{h}=1.45330\times 10^{-4},\\ J_{c}=-9.01772\times 10^{-5},\;J_{w}=-5.51531\times 10^{-5}. \end{array}$$

Using the unrounded numerical values, the first-law residual is below $10^{-17}$, confirming stationary energy conservation, while ${\dot{\Sigma }}_{C}=1.6023\times 10^{-4}>0$, consistent with the second-law Clausius condition. The minimum stationary eigenvalue is $2.37\times 10^{-7}$, while positivity is guaranteed by the GKSL form.

At the Redfield optimum, the corresponding currents are

(93) $$J_{h}=1.41650\times 10^{-4},\;J_{c}=-8.93628\times 10^{-5},\;J_{w}=-5.22876\times 10^{-5}.$$

Using the unrounded numerical values, the first-law residual is $2.5\times 10^{-17}$, while ${\dot{\Sigma }}_{C}=1.6351\times 10^{-4}>0$, again consistent with the second-law Clausius condition. The minimum stationary eigenvalue is $2.45\times 10^{-7}$. Thus the independent Redfield stationary state is positive in the reported regime despite the absence of a general complete- positivity guarantee.

## 7. Discussion

The comparison separates the robust physical statement from the model-dependent quantitative details. The robust statement is that the filter performs two opposing functions. Its Lorentzian spectrum suppresses the unwanted transition at $\omega_{c}$, while its finite damping and the scaling $g_{h}\propto \sqrt{\kappa }$ impose a transport cost when the filter is too narrow. Both explicit filter descriptions therefore generate an interior maximum, whereas the rate-only reduction cannot because it removes the slow filter degree of freedom.

The local GKSL and nonsecular Redfield calculations differ in how the residual baths resolve the interacting qutrit–filter system. The local GKSL equation couples the baths to the physical filter and bare qutrit transitions and has the important advantage of complete positivity. The Redfield calculation instead evaluates thermal rates at the dressed Bohr frequencies and retains near-degenerate cross-frequency terms. The shift from $\kappa_{★}/\Gamma_{h}=13.64$ to 7.79 is therefore a useful estimate of the quantitative sensitivity of the optimum to the dissipative construction, not a failure of the filtering mechanism.

The weak-coupling analysis gives additional guidance. Both retained dynamical descriptions connect continuously to the exact no-leak three-state engine, while a fully secular global construction does not. The latter result illustrates that complete positivity alone does not guarantee a uniform approximation when dressed splittings become nearly degenerate. Conversely, the Redfield comparison shows that the primary GKSL result is not merely an artifact of the local dissipators: the finite optimum and its systematic parameter trends persist when the baths are treated in the dressed basis.

The result should not be described as a generic benefit of “non-Markovianity.” The auxiliary mode produces a finite correlation time, but the relevant mechanism is more concrete: a physical frequency filter is also an element of the transport network. A phenomenological memory kernel or a stationary Lorentzian rate can reproduce spectral discrimination without reproducing the same dynamical bottleneck.

The zero-affinity terminal is likewise an operational energy repository rather than a microscopic battery. Replacing it by an explicit work-storage degree of freedom would sharpen the notion of extractable work [19], but is not required to identify the heat leak protection mechanism. Experimentally, filtering resonators and engineered electromagnetic environments provide a natural route to the regime considered here. The design rule is direct: the linewidth should resolve $\epsilon =\omega_{h}-\omega_{c}$ sufficiently well to suppress the parasitic transition while remaining large enough to sustain useful hot-side throughput.

### Possible Superconducting-Circuit Implementation

The finite-bandwidth protection mechanism considered here is well matched to present superconducting-circuit reservoir engineering platforms. Recent experiments provide two particularly relevant points of contact. Uusnäkki et al. demonstrated a cyclic quantum Otto engine using a flux-tunable transmon and a voltage-controlled quantum-circuit refrigerator (QCR) as an engineered thermal reservoir [20]. In a complementary recent experiment, an autonomous superconducting quantum heat engine was realized using coupled resonators interacting with spectrally filtered hot and cold reservoirs [13]. These experiments establish that tunable thermal microwave environments, auxiliary filtering resonators, and autonomous heat-to-work conversion are experimentally accessible in circuit QED.

The thermodynamic architecture studied here is nevertheless distinct from both implementations. The QCR experiment of Ref. [20] realizes sequential heating, cooling, and flux-control strokes of an Otto cycle. In the autonomous device of Ref. [13], distinct filtered hot and cold reservoirs act on a resonator-based working medium whose internal dynamics implement an approximate autonomous Otto cycle. Here, by contrast, the working medium is a stationary three-level system with simultaneously active hot, cold, and output channels. Most importantly, a *single* structured hot environment couples resonantly to the useful |g〉↔|e〉 transition and parasitically to the |i〉↔|e〉 transition. The proposed experiment therefore targets a different question: whether tuning the bandwidth of that hot environment can suppress a specific hot-to-cold thermodynamic shortcut without throttling the useful cycle.

**Figure 5:**
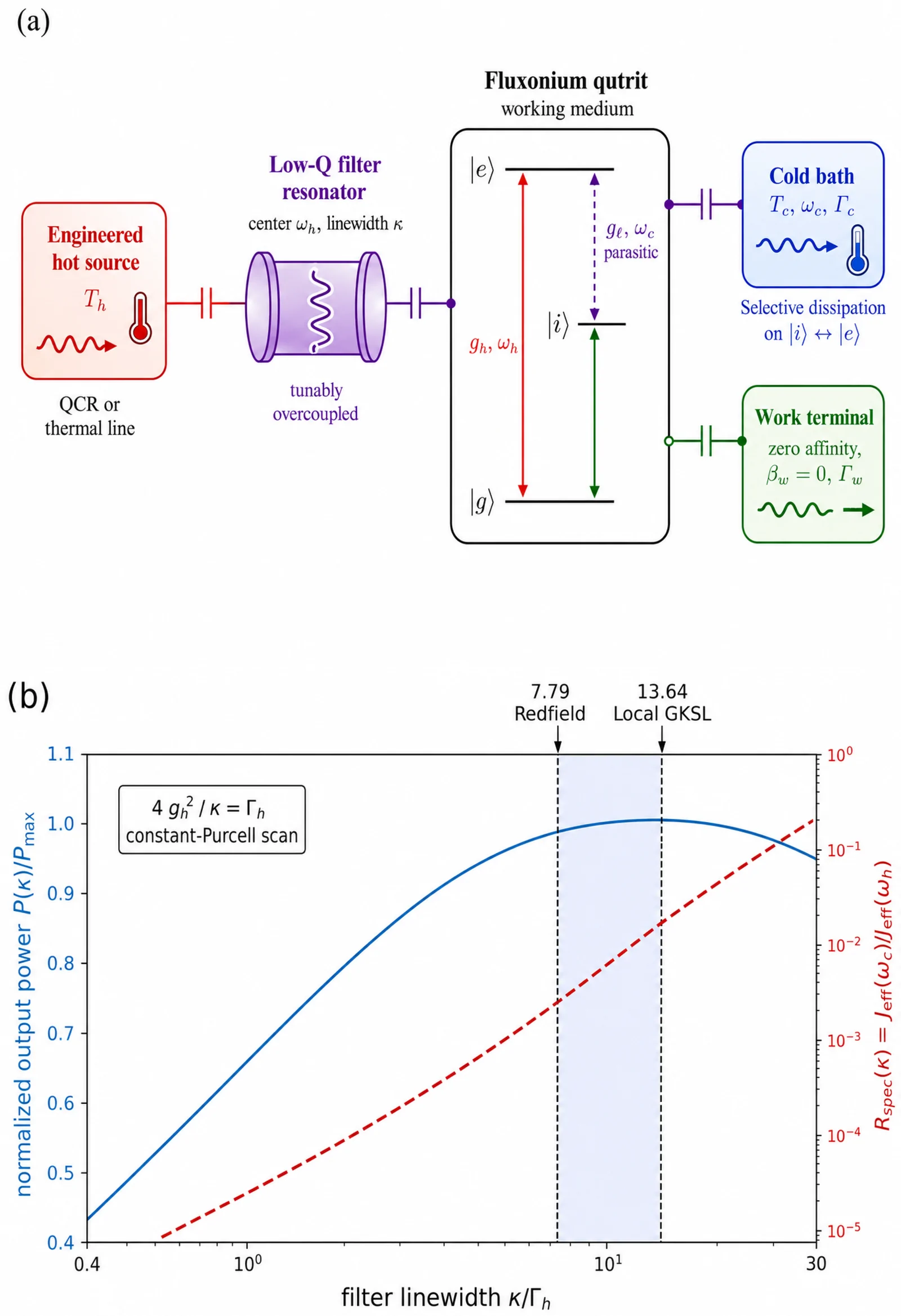
Proposed superconducting-circuit test of finite-bandwidth protection. (**a**) A strongly anharmonic three-level artificial atom, here represented by a fluxonium qutrit, is coupled to a tunable low-*Q* auxiliary resonator centered at $\omega_{h}$ with linewidth κ. The resonator acts as the structured hot filter and couples resonantly to the useful |g〉↔|e〉 transition with microscopic amplitude $g_{h}$, while coupling off-resonantly to the parasitic |i〉↔|e〉 transition with microscopic amplitude $g_{\ell }$. An engineered thermal microwave source supplies the hot reservoir, while independent channels provide selective cold dissipation on the |i〉↔|e〉 transition and the zero-affinity output terminal on |g〉↔|e〉, with $\epsilon =\omega_{h}-\omega_{c}$. (**b**) Experimental signature under a constant-Purcell scan, $4g_{h}^{2}/\kappa =\Gamma_{h}$. The normalized stationary output power (blue solid line) develops a maximum at finite linewidth, whereas the residual spectral-weight ratio $R_{\mathrm{spec}}(\kappa )\;=\;J_{\mathrm{eff}}(\omega_{c})/J_{\mathrm{eff}}(\omega_{h})\;=\;\kappa^{2}/\kappa^{2}+4\epsilon^{2}$ (red dashed line) increases monotonically with κ, corresponding to improved spectral rejection as the filter is narrowed. The shaded interval marks the representative optimum window bounded by the nonsecular Redfield and local-GKSL predictions, $\kappa_{★}^{R}/\Gamma_{h}\;=\;7.79$ and $\kappa_{★}^{\mathrm{GKSL}}/\Gamma_{h}\;=\;13.64$, respectively.

A natural implementation would use a strongly anharmonic superconducting artificial atom operated as an effective qutrit. Fluxonium is an attractive candidate because its level structure and selection rules can be engineered to access transition configurations that are unavailable in a nearly harmonic ladder; in particular, Λ-type three-level structures and otherwise forbidden transitions have been demonstrated experimentally [21]. The useful transition |g〉↔|e〉 would be coupled to a deliberately low-*Q* auxiliary microwave resonator of frequency $\omega_{h}$ and externally controlled linewidth κ. This resonator plays the role of the structured hot filter. The same resonator field also couples off resonantly to |i〉↔|e〉, thereby realizing the intended and parasitic matrix elements $g_{h}$ and $g_{\ell }$ of Equation (33). A separate frequency-selective dissipative channel coupled to the *i*–*e* transition provides the cold reservoir, while the *g*–*i* transition is connected to an output channel implementing the zero-affinity terminal used in the present stationary model. Figure 5 summarizes a possible superconducting-circuit realization of the finite-bandwidth protection mechanism and the corresponding experimental signature.

The experimentally relevant distinction between the rate-only and explicit filter descriptions can then be tested directly. According to Equation (49), the parasitic spectral weight decreases monotonically as the filter is narrowed. If the resonator is retained dynamically, however, both explicit filter descriptions predict that the output power eventually decreases for sufficiently small κ. The experimentally decisive signature is therefore the simultaneous observation of improving spectral rejection and a turnover of the stationary output power at a finite filter linewidth.

Representative physical scales can be obtained by choosing

(94) $$\omega_{h}/2\pi =6\;\mathrm{GHz},$$

while retaining the dimensionless parameters used throughout the calculations. This gives

(95) $$\omega_{c}/2\pi =3.6\;\mathrm{GHz},\;\epsilon /2\pi =2.4\;\mathrm{GHz},$$

and, from $\Gamma_{h}/\omega_{h}=0.01$,

(96) $$\Gamma_{h}/2\pi ≃60\;\mathrm{MHz}.$$

The two explicit filter calculations then locate the maximum-power bandwidth at approximately

(97) $$\kappa_{★}/2\pi ≃0.47\;\mathrm{GHz}\;(\mathrm{nonsecular}\;\mathrm{Redfield})$$

and

(98) $$\kappa_{★}/2\pi ≃0.82\;\mathrm{GHz}\;(\mathrm{local}\;\mathrm{GKSL}).$$

Equivalently, the predicted optimum lies in the representative window

(99) $$7.8≲\frac{\kappa_{★}}{\Gamma_{h}}≲13.6,$$

corresponding to resonator quality factors of order Q≃7–13. This interval should not be interpreted as universal; rather, it gives a realistic target range while also quantifying the sensitivity of the optimal linewidth to the treatment of the residual baths.

Under the constant-Purcell protocol of Equation (38), the corresponding coherent coupling strengths are of order

(100) $$g_{h}/2\pi ≃84–111\;\mathrm{MHz}.$$

The temperatures $T_{h}=0.5\;\omega_{h}$ and $T_{c}=0.2\;\omega_{h}$ translate to approximately 144mK and 58mK, respectively. These numbers are intended as representative circuit-QED scales rather than as a device-specific design.

A proof-of-principle experiment would therefore implement the constant-Purcell protocol of Equation (38) while sweeping the externally controlled filter linewidth κ. Implementing this protocol would require either a tunable qutrit–filter coupler or flux control of the relevant transition matrix element so that $g_{h}$ can be varied independently of the filter’s external damping. For each value of κ, one would measure the stationary output power together with the response of the hot channel at $\omega_{c}$. The characteristic signature would be that the parasitic spectral weight continues to decrease as κ is reduced, whereas the power reaches a maximum within a finite-bandwidth window and subsequently decreases. Such a measurement would directly distinguish the dynamical throughput cost of a physical filter from the purely spectral protection captured by an adiabatically eliminated rate model.

## 8. Conclusions

We have established an analytically grounded and numerically cross-validated picture of finite-bandwidth protection in a three-level quantum heat engine. The exact three-state network proves that the unwanted hot transition is a hot-to-cold short circuit and yields a closed threshold for engine reversal. An explicit damped filter then reveals the complementary cost of implementing spectral selectivity: an excessively narrow filter becomes dynamically slow.

The central finding is independent of a single open-system approximation. A completely positive local GKSL model and an independent nonsecular Redfield calculation both recover the exact no-leak Markovian engine in the appropriate weak-coupling limit and both predict a finite maximum-power bandwidth. For equal useful and parasitic microscopic coupling amplitudes they give, respectively, $\kappa_{★}/\Gamma_{h}=13.6443$ and 7.7938. The quantitative displacement of the optimum identifies the sensitivity to bath modeling, while the existence of the optimum, its high no-leak power retention, and its systematic shift with parasitic coupling are robust.

The resulting reservoir engineering principle is therefore specific: spectral filtering can protect a continuous quantum heat engine from a parasitic thermodynamic cycle, but a real filter cannot be narrowed without a dynamical cost. The useful operating point is set by the balance between spectral rejection and energy throughput.

## Figures and Tables

**Table 1:** Comparison of the descriptions for χ=1. The rate-only model has no interior optimum because the explicit filter response time has been eliminated. The fully secular global model is excluded from performance comparisons because it fails the weak-coupling recovery test of Figure 2a.

Method	$\kappa_{★}/\Gamma_{h}$	$P_{★}$	$\eta_{★}$
Local GKSL	13.6443	$5.5153\times 10^{-5}$	0.37950
Nonsecular Redfield	7.7938	$5.2288\times 10^{-5}$	0.36913
Lorentzian rates	κ→0	$5.9824\times 10^{-5}$	0.4000
Fully secular global	excluded	–	–

## Data Availability

The data generated and analyzed during this study are available from the corresponding author upon reasonable request.

## Appendix A. Vectorized Local-GKSL Stationary Equation

For column vectorization,

(A1) $$\mathrm{vec}\left(A\rho B\right)=\left(B^{T}⊗A\right)\mathrm{vec}\left(\rho \right).$$

The Hamiltonian contribution is

(A2) $$L_{H}=-i\left(I⊗H-H^{T}⊗I\right),$$

and one Lindblad jump operator contributes

(A3) $$D_{L}=L^{*}⊗L-\frac{1}{2}I⊗L^{†}L-\frac{1}{2}{\left(L^{†}L\right)}^{T}⊗I.$$

The full stationary Liouvillian is the sum of the Hamiltonian and all jump contributions. One row is replaced by the trace-normalization condition.

## Appendix B. Nonsecular Redfield Implementation

In the eigenbasis of *H*, Equation (58) is evaluated element by element at the actual Bohr frequency $E_{n}-E_{m}$. The superoperator corresponding to Equation (59) is then added to the Hamiltonian Liouvillian without frequency grouping or full secularization. The resulting stationary linear system is solved with the same trace replacement as in the local-GKSL calculation. Hermiticity is symmetrized only at numerical roundoff before stationary eigenvalues and currents are evaluated. For the weak-coupling calculation in Figure 2a, the Redfield ratios $P/P_{M}$ are 0.928327, 0.976988, 0.992123, 0.997568, 0.999138, 0.999689, and 0.999847 as Γ is reduced from $10^{-2}$ to $10^{-5}$; the corresponding local-GKSL values are 0.930703, 0.977731, 0.992374, 0.997644, 0.999163, 0.999697, and 0.999850.

## Appendix C. Fully Secular Global Control

For completeness, we specify the fully secular global construction used only as the diagnostic control in Figure 2. The starting point is the same enlarged qutrit–filter Hamiltonian *H* of Equation (33), which is diagonalized as

(A4) $$H|n\rangle =E_{n}|n\rangle .$$

The system coupling operators are the same as those used in the nonsecular Redfield calculation,

(A5) $$S_{h}=a+a^{†},\;S_{c}=\left|e\rangle \langle i\right|+|i\rangle \langle e|,\;S_{w}=\left|i\rangle \langle g\right|+|g\rangle \langle i|.$$

For each terminal ν∈{h,c,w} and each Bohr frequency ω, we introduce the dressed transition operator

(A6) $$A_{\nu }\left(\omega \right)=\sum_{E_{n}-E_{m}=\omega }\left|m\rangle \langle m\right|S_{\nu }|n\rangle \langle n|.$$

Thus, for ω>0, $A_{\nu }\left(\omega \right)$ lowers the energy of the enlarged system by ω. Transitions sharing the same Bohr frequency are retained within the same operator $A_{\nu }\left(\omega \right)$, while all terms involving distinct Bohr frequencies are discarded by the full secular approximation.

The corresponding global GKSL generator is

(A7) $$L_{\nu }^{\sec}\left[\rho \right]=\sum_{\omega }\gamma_{\nu }\left(\omega \right)\left[A_{\nu }\left(\omega \right)\rho A_{\nu }^{†}\left(\omega \right)-\frac{1}{2}\left\{A_{\nu }^{†}\left(\omega \right)A_{\nu }\left(\omega \right),\rho \right\}\right],$$

and the stationary state satisfies

(A8) $$0=-i\left[H,\rho_{\mathrm{ss}}\right]+\sum_{\nu =h,c,w}L_{\nu }^{\sec}\left[\rho_{\mathrm{ss}}\right].$$

No Lamb-shift contribution is included, consistently with the other stationary comparisons in the manuscript.

For the residual hot and cold reservoirs, we use the same frequency-resolved thermal rates as in the Redfield calculation,

(A9) $$\gamma_{\nu }\left(\omega \right)=\left\{\begin{array}{cc} \Gamma_{\nu }\left[n_{\nu }\left(\omega \right)+1\right],\; & \omega >0,\\ \Gamma_{\nu }n_{\nu }\left(|\omega |\right),\; & \omega <0, \end{array}\right.$$

where

(A10) $$n_{\nu }\left(\omega \right)=\frac{1}{e^{\omega /T_{\nu }}-1}.$$

For the residual hot bath one sets $\Gamma_{h}\to \kappa$, as in Equation (60). The work terminal is assigned the same flat zero-affinity rate used in the Redfield calculations,

(A11) $$\gamma_{w}\left(\omega \right)=\Gamma_{w}$$

on the energy-exchanging dressed transitions.

The essential difference from the nonsecular Redfield construction is therefore not the bath spectra, temperatures, or system Hamiltonian, but the removal of every cross-frequency contribution with $\omega \neq \omega^{\prime }$. Schematically, the Redfield generator contains terms of the form

(A12) $$A_{\nu }\left(\omega \right)\rho A_{\nu }^{†}\left(\omega^{\prime }\right),\;\omega \neq \omega^{\prime },$$

whereas Equation (A7) retains only $\omega =\omega^{\prime }$. The secular generator is consequently of GKSL form and completely positive.

This construction is normally appropriate when distinct Bohr frequencies are separated by scales large compared with the dissipative broadening. However, in the present weak-coupling limit, some dressed splittings collapse together with the overall coupling scale. Full secularization then removes cross-frequency terms before this limiting degeneracy is resolved. As shown in Figure 2a, the resulting stationary power does not approach the exact no-leak three-state Markovian value: in the weakest-coupling point shown,

(A13) $$\frac{P_{\sec}}{P_{M}}≃1.449,$$

whereas both the local-GKSL and nonsecular Redfield descriptions approach unity. We therefore use the fully secular construction only as a diagnostic of the near-degenerate secular approximation and not for the quantitative performance comparisons of Section 6.
